# Influence of Two Vaccination Campaigns on Genetic Diversity of Invasive *Neisseria meningitidis* Isolates in Northern Spain (1997–2008)

**DOI:** 10.1371/journal.pone.0008501

**Published:** 2009-12-30

**Authors:** Diego Vicente, Olatz Esnal, M. José López de Goicoechea, Ramón Cisterna, Emilio Pérez-Trallero

**Affiliations:** 1 Microbiology Service and Reference Laboratory for Meningococcal Infections of the Basque Country, Hospital Donostia, San Sebastián, Spain; 2 Biomedical Research Centre Network for Respiratory Diseases (CIBERES), San Sebastián, Spain; 3 Microbiology Service, Hospital Galdakao, Galdakao, Bizkaia, Spain; 4 Microbiology Service, Hospital Basurto, Bilbao, Spain; 5 Department of Preventive Medicine and Public Health, Faculty of Medicine, University of the Basque Country, San Sebastián, Spain; University of Witwatersrand, South Africa

## Abstract

**Background:**

*Neisseria meningitidis* diversifies rapidly, due to its high recombination rates. The aim of this study was to analyze the possible impact of two vaccination campaigns (a once-off A/C polysaccharide vaccination campaign in people aged 18 months to 20 years old in 1997, and a meningococcal C conjugate vaccination campaign in children aged ≤6 years old from 2000 to 2008) on diversification of the population of invasive isolates obtained between 1997 and 2008. All of the 461 available isolates were included (2, 319, 123, 11 and 6 belonging to serogroups A, B, C, Y and W-135, respectively).

**Methodology/Principal Findings:**

The isolates were analyzed for diversity using multilocus sequence typing, eBURST and the S.T.A.R.T.2 program. One hundred and seven sequence types (ST) and 20 clonal complexes were obtained. Five different STs (ST11, ST8, ST33, ST1163 and ST3496) included 56.4% of the isolates. With the exception of ST11, all other STs were associated with a specific serogroup. Epidemic circulation of serogroup C ST8 isolates was detected in 1997–1998, as well as epidemic circulation of ST11 isolates (serogroups B and C) in 2002–2004. The epidemic behavior of serogroup B ST11 (ST11_B:2a:P1.5) was similar, although with lesser intensity, to that of ST11 of serogroup C. Although clonality increased during epidemic years, the overall diversity of the meningococcal population did not increase throughout the 12 years of the study.

**Conclusion:**

The overall diversity of the meningococcal population, measured by the frequency of STs and clonal complexes, numbers of alleles, polymorphic sites, and index of association, remained relatively constant throughout the study period, contradicting previous findings by other researchers.

## Introduction

The only natural reservoir for *Neisseria meningitidis* is the human pharynx. Persons with nasopharyngeal colonization become asymptomatic carriers. *Neisseria meningitidis* clones are considered to diversify rapidly due to the high rates of recombination and mutation of this bacterium [1]. Diversification is much lower among isolates involved in disease episodes than in those found in asymptomatic carriers [2], although isolates causing invasive disease show wide variation in space and time [3]. It is generally accepted that host immune pressure leads to antigenic diversity. Polysaccharide vaccines are effective in controlling outbreaks caused by meningococci of the serogroups included in the vaccine but do not induce long-term protection, herd immunity, or protection in children aged less than 2 years old. The recent introduction of the meningococcal conjugate vaccine has reduced both the incidence of meningeal disease and pharyngeal colonization [4]–[6]. Despite the benefits of vaccination, other effects such as capsule switching are a cause of concern [7], [8], [9]. In addition, the decrease in meningococcal circulation and pharyngeal colonization after mass immunization may generate a niche that could be occupied by other isolates, which may contribute to an increase in diversity [10].

The aim of this study was to identify the distribution of clones and the diversity of disease-associated isolates of *Neisseria meningitidis* collected over a 12-year period in a region of northern Spain (the Basque Country) where two mass immunization campaigns have been carried out in the last few years.

## Materials and Methods

All isolates causing invasive meningococcal disease in the provinces of Araba, Gipuzkoa and the eastern region of Bizkaia in the Basque Country, northern Spain, were included. A total of 461 isolates from blood or cerebrospinal fluid from patients with meningococcal disease between January 1997 and December 2008 were available for full molecular characterization. Only one isolate per patient was included.

Capsular serogroup was identified through latex agglutination with group-specific capsular polysaccharides of serogroups A, B, C, W135 and Y (Murex Biotech Ltd., Dartford, England). In non-serogroupable isolates, genogroup specific real-time polymerase chain reaction (PCR) was performed with a LightCycler apparatus (Roche Diagnostics Mannheim, Germany), using primers (Tib Molbiol, Berlin, Germany) specific for the *siaD* and *orfA* genes [11].

The genosubtype was determined as previously described [12] through amplification of two fragments of the *porA* gene coding for VR1 and VR2, sequencing, and subsequent comparison of the deduced amino acid sequence with PorA types available at the *N. meningitidis* PorA variable region database (http://neisseria.org/nm/typing/pora/).

Multilocus sequence typing (MLST) was performed by amplification and sequencing of seven housekeeping genes [13]. Briefly, a suspension containing approximately 10^6^ cells was incubated with 0.1 mg/mL proteinase K. Genomic DNA was extracted from all isolates with a QIAamp DNA Blood Mini kit (QIAGEN, Hilden, Germany). PCRs were carried out with 50 µL amplification mixtures using 5–50 µg of template DNA in a thermal cycler (Applied Biosystems, Foster City, CA, USA). The seven amplicons obtained were sequenced (forward strand or forward and reverse when the analyzed sequence did not match the reference sequences) in an ABI PRISM 3100 Genetic Analyzer (Applied Biosystem, Foster City, CA, USA). Primers, determination of sequence alleles, and designation of sequence types (ST) are described on the MLST web site (http://neisseria.org/nm/typing/mlst/). The clonal complexes reported were those contained in the PubMLST database of the above-mentioned MLST website.

Pulsed-field gel electrophoresis (PFGE) was performed according to previously described standard protocols [14] using *Spe*I restriction endonuclease (Amersham Pharmacia Biotech, Buckinghamshire, England). PFGE patterns were analyzed with the Diversity Database Fingerprinting software version 2 (BioRad, Hercules, USA) and a dendrogram was constructed by the unweighted pair group method with arithmetic averages, the Dice coefficient, and a position tolerance of 1%. Isolates with a PFGE pattern similarity of ≥85% were considered to represent a clone.

### Diversity Analysis

eBURST is a tool that allows STs to be classified into distinct groups or lineages according to their similarity in allelic profiles. Every ST within an eBURST group has a minimum number of identical alleles in common with at least one other ST in the group. In this study, the eBURST analysis was performed with the stringent (default) group definition of six out of seven shared alleles, as described at http://eburst.mlst.net/. Use of this criterion (6/7 shared alleles) ensures that the isolates classified within a group belong to the same clonal complex.

Intra- and intergroup comparisons were made using the Sequence Type Analysis and Recombinational Tests (S.T.A.R.T.2) [15] suite of programmes available at http://pubmlst.org/software/analysis/start2/. The S.T.A.R.T. programme allows group assignment to be performed in bacterial populations and recombination and selection phenomena to be analyzed using multilocus sequence data.

The parameters used to measure diversity over the study years were as follows: serogroup frequencies, STs and clonal complexes and their respective ratios with respect to the number of isolates, the number of alleles present at each locus, the number of polymorphic sites at each locus (positions at which variation was observed) and their respective ratios with respect to the number of isolates. Finally the index of association (*I_A_*) was calculated. This test allows the degree of recombination within a population to be quantified through the formula: I_A_ = Vo/Ve-1 (where Vo is the variation observed in the number of loci in which two individuals differ and Ve is the expected variation). *I_A_* = 0 indicates frequent recombination events and *I_A_*>0 indicates increasing clonality due to a lack of recombination events.

The phenotypic and genotypic characteristics of isolates representing each of the STs reported in this study were deposited in the PubMLST Database (http://pubmlst.org/neisseria/).

### Immunization Campaigns

Two mass immunization campaigns were carried out in the Basque Country in the last 12 years (1997–2008). The first was a once-off campaign in October 1997, using the bivalent A/C meningococcal polysaccharide vaccine (Mencevax®, GSK, Tres Cantos-Madrid, Spain). The target population consisted of 414,888 persons aged 18 months to 20 years with an overall coverage of 88.6% (99% in children and teenagers aged 6–17 years and 58% in 18–20-year-olds). Three years later, in October 2000, the monovalent meningococcal serogroup C conjugate vaccine (MCVc) (Meningitec®, Wyeth Farma, San Sebastian de los Reyes-Madrid, Spain) was introduced in the Basque Country. This late vaccination campaign introduced a routine three-dose infant vaccination series and included a mass catch-up, A total of 78.2% of the children born in 2000 received the three vaccine doses. The catch-up was made in 2000 in children aged from 12 months to 6 years, with a coverage of 95%. More than 94% of children born since 2001 have received three vaccine doses.

To calculate the incidence rates of invasive meningococcal disease by age groups, official data from the population living in the study areas (Eustat, Basque Institute office; http://www.eustat.es) were used. Census data were available for 1996, 2001 and 2006, while the values for the remaining years were estimated.

Epidemic years are defined as those in which the incidence of meningococal disease increased due to the predominance of a single ST

### Statistical Analysis

The Chi square test for trends was used to compare the annual incidence rate. To analyze differences in clonality, ratios were compared (Cramér-von Mises) with calculation of P-values through permutations (10,000 random permutations).

### Ethics Statement

The creation of the “Meningococcal Infection Reference Laboratory” in the 90s had its justification in the possibility of analyzing the phenotype and genotype of strains causing invasive infections in our region, to gain knowledge about the epidemiology of the disease. In the present study no human experimentation was conducted, with all studies carried out on microorganisms. The data referred to are associated with meningococcal strains, with no patient information used other than age, gender and vaccination status. Publication of the results obtained by this Reference Laboratory concerning the phenotype and genotype of the strains studied and their relation to age, gender and vaccination status of the patients was approved by the ‘Comité Ético de Investigación Clínica del Área Sanitaria de Gipuzkoa’.

## Results

Of the 497 microbiologically-confirmed cases of invasive meningococcal disease in the study area, 349 (70.2%) were caused by serogroup B meningococci, 129 (26%) by serogroup C (Table 1), 11 (2.2%) by serogroup Y, 6 (1.2%) by serogroup W-135, and 2 (0.4%) by serogroup A. The mean annual number of cases was 41 (range 23–59), giving a mean annual incidence of 3.6×100,000 inhabitants. In 461 (92.8%) of these 497 episodes, the strain for molecular study of diversity was available (Table 2). After the introduction of the MenC conjugate vaccine (in October 2000), 224 episodes caused by serogroup B and 72 by serogroup C were detected, of which 93 (41.5%) and 11 (15.3%), respectively, occurred in children eligible for vaccination, four of whom were previously vaccinated.

**Table 1:** Number of cases and incidence rate (in brackets) of invasive *Neisseria meningitidis* of serogroup B and C by groups of age in the Basque Country, north of Spain (1997–2008).

	<1 year				1–6 years				7–12 years				13–18 years				>18 years				Total			
	B		C		B		C		B		C		B		C		B		C		B		C	
**1997**	0^a^	(0.0)^b^	3	(34.6)	4	(7.4)	12	(22.1)	1	(1.4)	5	(6.9)	1	(1.0)	4	(3.9)	6	(0.6)	5	(0.5)	13	(1.1)	28	(2.3)
**1998**	2	(23.1)	0	(0)	18	(33.1)	5	(9.2)	4	(5.5)	1	(1.4)	0	(0)	1	(1.0)	8	(0.8)	5	(0.5)	32	(2.7)	12	(1.0)
**1999**	4	(46.2)	0	(0)	12	(22.1)	1	(1.8)	3	(4.2)	0	(0)	2	(2.0)	0	(0)	10	(1.0)	1	(0.1)	31	(2.6)	2	(0.2)
**2000**	7	(80.8)	2	(23.1)	16	(29.4)	8	(14.7)	1	(1.4)	0	(0)	1	(1.0)	0	(0)	4	(0.4)	5	(0.5)	29	(2.4)	15	(1.3)
**2001**	3	(34.6)	1	(11.5)	9	(16.6)	0	(0)	2	(2.8)	1	(1.4)	3	(3.0)	2	(2.0)	11	(1.2)	3	(0.3)	28	(2.3)	7	(0.6)
**2002**	4	(46.2)	0	(0)	5	(9.2)	4	(7.4)	1	(1.4)	3	(4.2)	6	(5.9)	3	(3.0)	15	(1.6)	18	(1.9)	31	(2.6)	28	(2.3)
**2003**	4	(46.2)	0	(0)	13	(23.9)	2	(3.7)	2	(2.8)	1	(1.4)	8	(7.9)	4	(3.9)	12	(1.3)	8	(0.8)	39	(3.3)	15	(1.3)
**2004**	2	(26.3)	0	(0)	6	(12.6)	1	(2.1)	3	(4.7)	0	(0)	6	(6.7)	6	(6.7)	15	(1.8)	3	(0.4)	32	(3.1)	10	(1.0)
**2005**	3	(39.4)	0	(0)	14	(29.3)	0	(0)	4	(6.3)	0	(0)	6	(6.7)	1	(1.1)	10	(1.2)	3	(0.4)	37	(3.5)	4	(0.4)
**2006**	5	(65.7)	0	(0)	7	(14.7)	0	(0)	2	(3.2)	0	(0)	3	(3.4)	0	(0)	11	(1.3)	2	(0.2)	28	(2.7)	2	(0.2)
**2007**	5	(65.7)	0	(0)	14	(29.3)	1	(2.1)	1	(1.6)	0	(0)	3	(3.4)	0	(0)	7	(0.8)	1	(0.1)	30	(2.9)	2	(0.2)
**2008**	4	(52.5)	0	(0)	3	(6.3)	1	(2.1)	2	(3.2)	0	(0)	2	(2.2)	1	(1.1)	8	(1.0)	2	(0.2)	19	(1.7)	4	(0.4)
**Total**	43	(43.6)	6	(6.1)	121	(19.5)	35	(5.7)	26	(3.2)	11	(1.3)	41	(3.6)	22	(1.9)	117	(1.1)	56	(0.5)	349	(2.6)	129	(0.9)
**X^2^ for trend**		0.3		6.8		1.2		25.3		0.2		8.1		1.9		0.2		1.9		2.7		0		21.9
**P value**		NS		0.009		NS		<0.001		NS		0.004		NS		NS		NS		NS		NS		<0.001

a number of cases.

b incidence per 100,000 inhabitants.

NS: Difference not significant.

MLST analysis of the isolates showed 107 different STs. Of these, 54 were new STs, not previously been described in the PubMLST Database (http://pubmlst.org/neisseria/submission.shtml). The distribution of isolates by ST showed wide heterogeneity. The predominant ST was ST11, representing 23% (106/461) of the isolates, and more than half of the isolates (260/461, 56.4%) were included in five STs: ST11, ST8 (51 isolates), ST33 (47 isolates), ST1163 (37 isolates), and ST3496 (19 isolates). In contrast, 77 isolates each represented a distinct ST (77 STs with a single isolate). The number of STs detected each year ranged from 11 (1997 and 2007) to 29 (2003) (Table 2). The ratio between the number of isolates and the number of STs was not constant over time, being significantly lower in some years than others (P<0.001). However, this ratio showed no upward or downward trend over the twelve years of study.

**Table 2:** Serogroup, sequence type and clonal complex yearly frequency among 461 available invasive meningococci from the Basque Country, north of Spain (1997–2008).

	1997	1998	1999	2000	2001	2002	2003	2004	2005	2006	2007	2008
N° of isolates	41	44	34	42	30	60	55	38	39	25	31	22
**Serogroup** 1												
B	12(29%)	31(71%)	31(91%)	27(64%)	20(67%)	31(51.5%)	37(67%)	27(71%)	33(85%)	22(88%)	29(94%)	19(86%)
C	27(66%)	11(25%)	2(5.5%)	14(34%)	7(23%)	27(45%)	15(27%)	9(24%)	4(10%)	2(8%)	2(6%)	3(14%)
**N° of STs** [new STs]^2^ (ratio isolates/STs)	11[1] (3.73)	19[3] (2.32)	23[10] (1.48)	23[6] (1.83)	14[1] (2.14)	19[6] (3.16)	29[15] (1.9)	15[7] (2.53)	19[4] (2.05)	13[2] (1.92)	11[1] (2.8)	13[2] (1.69)
**N° clonal complexes** (ratio^3^ isolates/clonal complexes)	7 (5.86)	8 (5.50)	11 (3.09)	11 (3.82)	10 (3)	9 (6.67)	12 (4.58)	10 (3.8)	11 (3.55)	9 (2.78)	7 (4.4)	7 (3.1)
**N° alleles [mean]** (ratio isolates/alleles)	7 (5.86)	9 (4.89)	9 (3.78)	10 (4.2)	9 (3.33)	9 (6.67)	12 (4.58)	8 (4.75)	10 (3.9)	9 (2.78)	7 (4.43)	8 (2.75)
**N° polymorphic sites [mean]**	45	56	58	59	57	45	60	46	57	60	57	54
Index of association *(I_A_)* 4	5.6493	4.2209	2.3128	3.4481	4.4125	4.9277	4.2530	4.6870	3.5411	4.2722	4.5476	3.4451

1 Serogroups A (n = 2), Y (n = 11) and W-135 (n = 6) are not represented in the table.

2 New STs described in this article.

3 Ratio: the higher the value, the lower the diversity (greater clonality)

4 *I_A_*: indicates the frequency of recombination events. *I_A_* = 0 indicates frequent recombination events and *I_A_*>0 indicates increasing clonality due to a lack of recombination events.

There was a strong association between ST and serogroup. All ST32, ST33 and ST1163 isolates expressed serogroup B, while 50 of the 51 ST8 isolates expressed serogroup C. Except for three ST23 isolates (one each from serogroups B, C and Y), and ST11 (reported in greater detail below), the remainder included only or mainly (>85%) isolates from a single serogroup: ST22 (serogroup W135), ST1768 (serogroup Y), and the remaining STs (serogroup B).

The 319 serogroup B isolates were grouped into 94 STs, while the 123 serogroup C isolates belonged to only 12 different STs (Table 3). Isolates expressing serogroup B showed higher ST diversity (ratio 3.4) than those expressing serogroup C (ratio 10.3) (P<0.001). ST diversity was obtained by applying the ratio between the number of isolates and the number of STs; the lower the value of the ratio, the greater the diversity.

**Table 3:** Distribution by clonal complex, sequence type and PorA type of 319 serogroup B and 123 serogroup C invasive meningococci from the Basque Country, north of Spain (1997–2008).

Serogroup	ST clonal complex (No isolates)	Sequencetype (ST), PorA type (P) (No isolates)
C	ST11/ET-37 complex (64)	**ST11, P5-10 (58)**; ST11, P5-2 (4); ST11, P5-14 (1); ST3419, P5-10 (1)
C	ST8/Cluster A4 (55)	**ST8, P5-2 (49)**; ST8, P5-12 (1);ST66, P5-2 (1); ST3331, P5-2 (1); ST6414, P5-2 (1); ST6420, P5-2 (1); ST6645, P5-2 (1)
C	ST461 complex (1)	ST461, P5-2 (1)
C	ST213 complex (1)	ST3496, P22-14 (1)
C	ST23 complex/ClusterA3 (1)	ST23, P5-10 (1)
C	Not clonal complex (1)	ST6411, P21-4 (1)
B	ST32/ET-5 complex (78)	**ST33, P5-2 (23)**; **ST33, P19-15 (19)**; ST33, P7-4 (2); ST33, P18-3 (1); ST33, P19-2 (1); **ST749, P19-15 (12);**ST749, P5-15 (1); ST749, P21-15 (1); ST32, P7-16 (2)ST32, P7-4 (1); ST32, P19-15 (1); ST32, P19-16 (1); ST32, P17-13 (1); ST34, P19-15 (4); ST343, P7-16 (1); ST639, P7-16 (1); ST5101, P19-15 (1); ST5682, P19-15 (1); ST6435, P13-12 (1); ST6640, P7-4 (1); ST6647, P5-2 (1); ST6979, P19-15 (1)
B	ST11/ET-37 complex (49)	**ST11, P5-10 (42)**; ST11, P5-2 (1); ST6648, P12 (2); ST3419, P5-10 (1); ST3419, P7-30 (1); ST4091, P5-10 (1); ST4706, P5-10 (1)
B	ST269 complex (n = 49)	**ST1163, P22-9 (33)**; ST1163, P22-14 (1); ST1163, P22-4 (1); ST1163, P7-30 (1); ST1163, P19-14 (1); ST269, P15-9 (1); ST5329, P21-16 (1); ST6409, P22-9 (1); ST6410, P5-10 (1); ST6416, P22-9 (1); ST6418, P22-9 (1); ST6422, P5-2 (1); ST6426, P22-9 (1); ST6428, P22-9 (1); ST6436, P22-9 (1); ST6637, P19-15 (1); ST6642, P14 (1)
B	ST41/44 Lineage 3(38)	**ST41, P7-4 (10)**; ST3752, P22-9 (6); ST3752, P7-30 (1); ST1947, P7-14 (4); ST409, P18-34 (2); ST6408, P18-10 (1); ST6408, P18-34 (1); ST44, P7-4 (1); ST571, P7-4 (1); ST1465, P12-9 (1); ST2820, P15-9 (1); ST3935, P5,2 (1); ST6075, P18-34 (1); ST6406, P18-25 (1); ST6413, P12-9 (1); ST6417, P7-4 (1); ST6429, P22-14 (1); ST6641, P12-9 (1); ST6646, P7-14 (1); ST6649, P18-9 (1);
B	ST461 complex (26)	ST461, P19-13 (9); ST461, P19-15 (2); ST461, P17-9 (1); ST461, P17-16 (1); ST461, P18-30 (1); ST1946, P19-13 (3); ST3494, P19-13 (2); ST6044, P,19-13 (2); ST2299, P19-13 (1); ST6415, P19-13 (1); ST6430, P19-13 (1); ST6431, P17-9 (1); ST6639, P5-2 (1)
B	ST213 complex (23)	**ST3496, P22-14 (17)**; ST3496, P5-10 (1); ST7309, P22-14 (2); ST213, P22-14 (1); ST3113, P22-14 (1); ST7307, P22-14 (1)
B	ST162 complex (13)	**ST162, P7-4 (11)**; ST6980, P7-4 (1); ST7308, P7-4 (1)
B	ST35 complex (11)	ST35, P22-14 (7); ST457, P22-14 (1); ST807, P22-14 (1); ST4418, P22-14 (1); ST6978, P22-14 (1)
B	Other clonal complex (11): ST865 complex (3); ST60 complex (1); ST8/Cluster A4 (1); ST 22 complex (1); ST 23 complex (1); ST 103 complex (1); ST 116 complex (1); ST 254 complex (1); ST 364 complex (1)	ST1306, P21 (1); ST4237, P21-16 (1); ST6644, P4 (1); ST60, P5-2 (1); ST8, P5-2 (1); ST22, P18-3 (1); ST23, P5-10 (1); ST6638, P7-4 (1); ST6643, P19-13 (1); ST6433, P22-14 (1); ST3216, P14 (1)
B	Not clonal complex (21)	ST4954, P19-15 (5); ST4954, P19-13 (1); ST6412, P5-2 (2); ST6412, P5-10 (1); ST6424, P19-15 (2); ST6421, P19-15 (1); ST6423, P19-15 (1); ST6651, P19-15 (1); ST1768, P22-14 (1); ST2196, P21-14 (1); ST6432, P22-14 (1); ST1572, P7-14 (1); ST6434, P7-14 (1); ST6977, P7-14 (1); ST7306, P19-15 (1)

**Bold** **type** indicates sequence types represented by 10 or more isolates.

Using the *Neisseria* PubMLST Database, 431/461 (93.5%) isolates and 89/107 (83.2%) STs detected in the Basque Country were assigned to one of the clonal complexes available in this database. In total, isolates belonging to 20 distinct clonal complexes were identified, the most numerous being the following: ST11/ET-37 complex (113 isolates), ST32/ET-5 complex (79 isolates), ST8/Cluster A4 (56 isolates), ST269 complex (49 isolates), ST41/44 Lineage 3 (38 isolates) and ST461 complex (27 isolates). These top six clonal complexes make up 78% of all the isolates.

Using eBURST, 71 STs (411 isolates) of the 107 STs identified in our region were classified into groups, with the remaining 36 being unrelated or singleton STs (50 isolates) (Table 4). Two of the groups identified could not be included in any of the clonal complexes defined to date (groups 3 and 5). Likewise, 24 singleton STs are described for the first time in this study. The number of STs in each group ranged from 2 to 11 (median 5) and the number of isolates from 2 to 111 (median 10). Group 3 was composed of 10 isolates, all serogroup B, belonging to four distinct STs (ST4954, ST6423, ST6424, ST6651), and group 5 by 9 isolates (5 serogroup Y, 3 serogroup B, 1 serogroup C) belonging to 5 STs (ST1768, ST2196, ST6407, ST6411, ST6432).

**Table 4:** eBURST analysis of 461 invasive meningococci from the Basque Country, north of Spain (1997–2008).

eBURST (No isolates)	STs^1^	Clonal Complex	Representative Phenotype^2^
Group 1 (n = 47)	1163(37); 269(1); **6409** 3(1); **6416**(1); **6418**(1); **6422**(1); **6426**(1); **6428**(1); **6436**(1); **6637**(1); **6642**(1)	ST269 complex	B:NT^4^:9
Group 2 (n = 28)	41(10); 3752(7); **6408**(2); 409(2); 44(1); 571(1); 2820(1); 6075(1); **6406**(1); **6429**(1); **6649**(1)	ST41/44 complex/Lineage 3	B:4:14
**Group 3** (n = 10)	4954(6); **6424**(2); **6423**(1); **6651**(1)	Non-defined	B:1:15
Group 4 (n = 76)	33(47); 749(14); 32(6); 34(4); 343(1); 639(1); 5101(1); 5682(1); **6647**(1)	ST32/ET-5 complex	B:4:15
**Group 5** (n = 9)	1768(4); **6407**(2); 2196(1); **6411**(1); **6432**(1)	Non-defined	Y:15:4
Group 6 (n = 25)	461(15); 1946(3); 3494(2); 6044(2); 2299(1); **6430**(1); **6431**(1)	ST461 complex	B:1:NT
Group 7 (n = 56)	8(51); 66(1); 3331(1); 6414(1); **6420**(1); **6645**(1)	ST8 complex/ClusterA4	C:2b:5,2
Group 8 (n = 24)	3496(19); 213(1); 3113(1); **7309**(2); **7307**(1)	ST213 complex	B:1:14
Group 9 (n = 111)	11(106); 3419(3); 4091(1); 4706(1)	ST11/ET-37 complex	C:2a:5/B:2a:5
Group 10 (n = 2)	1306(1); **6644**(1)	ST865 complex	-
Group 11 (n = 10)	35(7); 457(1); 807(1); 4418(1)	ST35 complex	B:4:14
Group 12 (n = 13)	162(11); **6980**(1); **7308**(1)	ST162 complex	B:NT:4
Singletons (n = 50)	22(7); 1947(4); 23(3); **6412**(3); **6648**(2); 60(1); 1465(1); 1572(1); 2394(1); 3216(1); 3544(1); 393(1); 4237(1); 5329(1); **6410**(1); **6413**(1); **6415**(1); **6417**(1); **6419**(1); **6421**(1); **6425**(1); **6427**(1); **6433**(1); **6434**(1); **6435**(1); **6638**(1); **6639**(1); **6640**(1); **6641**(1); **6643**(1); **6646**(1); **6650**(1); **6977**(1); **6978**(1); **6979**(1); **7306**(1)	-	-

1 ST: Sequence types in order of frequency (the number of isolates is given in brackets).

2 The representative phenotype was the phenotype expressed by most (>50%) of the strains in the group.

3 **In bold** the new groups and singletons described in this article.

4 NT: Non-typable.

Intra- and intergroup diversity analyzed with the S.T.A.R.T.2 programme showed that the allele frequency at each locus for each of the 12 years ranged from 4 for adk (1999, 2000, 2001, 2003, 2006 and 2007) to 15 for aroE and pgm in 2003. The number of polymorphic sites present at each locus ranged from 9 for adk (1999, 2000, 2001, 2003, 2006 and 2007) to 164 for aroE in 2000. Table 2 shows the annual mean and the ratio between each of the above-mentioned parameters and the number of isolates, as well as the *I_A_*.

The circulation of ST11 isolates deserves special mention since, of the 106 isolates of this ST, 43 expressed serogroup B and 63 expressed serogroup C capsules, all showing a similarity of >85% when analyzed by PFGE. Serotyping and porA VR typing revealed that all strains showed the 2a:P.1.5 phenotype with variations in VR2 except for one serogroup B strain, which, instead of serotype 2a, showed serotype 4.

**Figure 1:**
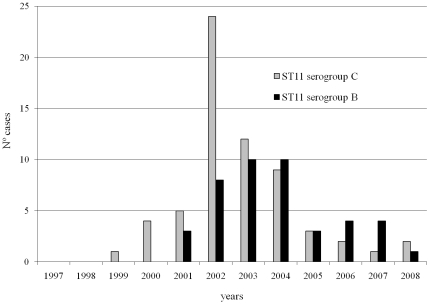
Annual distribution of serogroup ET15 variant of the ST11/ET37 complex. Annual distribution of serogroup B (n = 43) and C isolates (n = 63) belonging to the ET15 variant of the ET37 complex showing multilocus sequence type 11.

Complementary analysis of the *fum* gene allowed the 106 ST11 isolates to be classified within the ET15 variant of the ST11/ET37 clonal complex. The number of ST11 strains isolated each year and their corresponding serogroup are shown in Figure 1.

**Figure 2:**
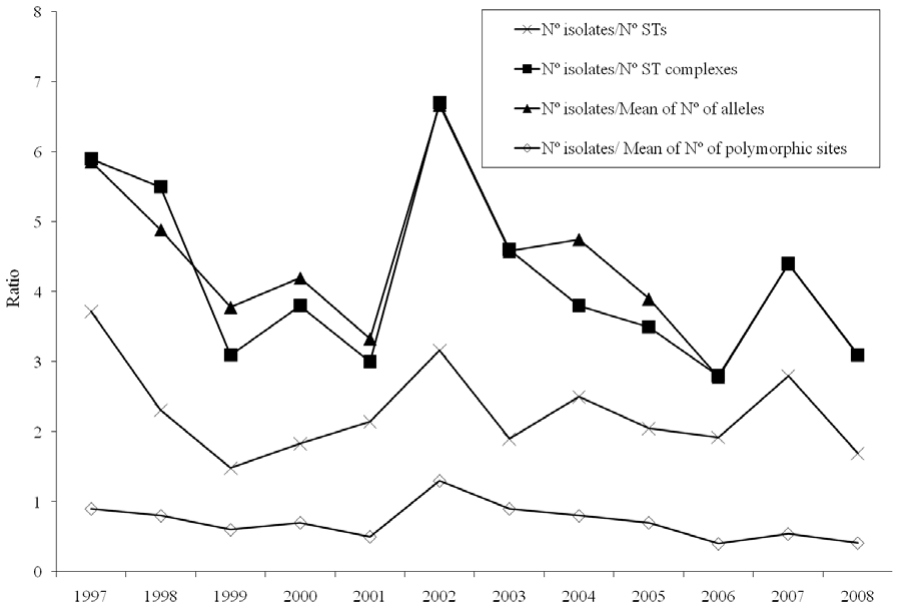
Diversity of the meningococcal population in the Basque Country, north of Spain, 1997–2008. The ratio between the number of isolates and four parameters (number of sequence types [ST], clonal complexes, alleles and polymorphic sites) is shown among 461 invasive meningococci.

During the study period, epidemic circulation of serogroup C ST8 isolates was detected in 1997–1998 and preferential circulation of ST11 isolates (serogroups B and C) was found in 2002–2004. Figure 2 indicates how clonality increased during epidemic years and how the diversity of the invasive meningococcal population in our region did not increase throughout the 12 years of the study.

## Discussion

Phenotypic and genotypic analysis was performed in all of the 461 available invasive meningococci isolates collected from the study area. These isolates came from a well delimited area in which active follow-up was performed of the episodes occurring in 12 consecutive years, 8 of these after vaccination with the meningococcal C conjugate vaccine was implemented in the population aged less than 7 years.

Despite the high capacity for genetic recombination shown by *N. meningitidis*, some disease-causing meningococcal clones or groups of related clones (clonal complexes) circulate practically unaltered all over the world for very prolonged periods. In the present study, circulation of invasive isolates belonging to more than 100 distinct STs was detected, although more than half of the isolates were grouped in only five STs, corresponding to the following clonal complexes: ST11/ET-37 complex, ST32/ET-5 complex, ST8 complex/Cluster A4, ST269 complex, and ST213 complex. This epidemiological situation is broadly similar to that in other areas of Europe and the developed world, both in countries that have included the conjugate vaccine in their immunization schedules and those that have not [16]–[20]. The data for 1999–2006 collected in 18 European countries through the EU-MenNet Project also showed wide variation in the STs among the invasive isolates analyzed, the predominant clonal complexes coinciding with those detected in our region [16].

The overall incidence of meningococcal disease showed no significant variation in tendency throughout the 12 years of the study, nor was significant variation observed in the incidence of disease caused by serogroup B meningococci. However, the incidence of disease caused by serogroup C *N. meningitidis* showed a decreasing tendency in children aged less than 12 years throughout the study period. Serogroup C caused 27% of the episodes, peaking in 2002 when this serogroup caused 45% (27/60) of the episodes, even though immunization in children aged less than 7 years old had been introduced 2 years previously and a vaccination coverage of ≥90% had been achieved in this age group. Most serogroup C cases in 2002 (18/27) occurred in adults >19 years old. Among the remaining 9 patients, only one 4-year-old child had received a dose of the conjugate vaccine. Several countries have reported a dramatic reduction in the incidence of meningococcal disease due to serogroup C isolates after the introduction of the conjugate vaccine in both vaccinated and non-vaccinated individuals [4], [5], [17]. In our region, vaccination achieved optimal protection among vaccinated individuals but did not curtail circulation of serogroup C isolates among non-vaccinated persons, especially in 2000 and 2003, when the ST11/ET37 complex circulated widely, as in other countries such as France, Iceland and the Czech Republic [16], [17], [19]. Comparison of the incidence of invasive diseases in children less than 7 years old in the study region between the prevaccination -conjugate vaccine- period (1997–1999) and the postvaccination period (2001–2008), revealed a highly significant reduction, due to a marked decrease in serogroup C cases (incidence rate 11.1 vs. 2.0 cases×100,000 inhabitants, respectively; P<0.001). Nevertheless, no differences in incidence were observed between the two periods for serogroup B cases in children aged less than 7 years old, or in any other age group.

In the Basque Country in 1997, a once-off vaccination campaign was performed with the polysaccharide vaccine in 18 months to 20-year-olds, achieving coverage of 88.6%. This polysaccharide vaccine and/or the subsequent conjugate vaccine could have had some effect on the circulation of ST11_B:2a:P1.5 isolates in the ensuing years. After 2001 meningococci of the ET15 variant of the ST11/ET37 complex, belonging to serogroup B, were increasingly isolated, matching the prevalence of serogroup C isolates of this clonal complex, which were already circulating. Strains of this ET15 variant circulated throughout the world, especially after 1990, causing major outbreaks [16]–[20]. The increase observed in the number of ST11_B:2a:P1.5 isolates was initially interpreted as capsule switching phenomenon, arising from the initial presence of ST11_C:2a:P1.5 [8]. Nevertheless, independently of whether the capsule switching phenomenon was stimulated or not by vaccination, the number of ST11_B:2a:P1.5 isolates initially increased and later decreased. This epidemic behavior was similar, although with lesser intensity, to that of the most typical isolates of ST11 of serogroup C.

The increase and subsequent decrease in the number of cases of both serogroups (Figure 1), as well as their superposition (serogroup B plus serogroup C), reflects the epidemic dissemination of the hypervirulent ST11 in an only partially immunized population. The experience in England and Wales, where administration of the meningococcal C conjugate vaccine was broadened to include persons up to 19 years old, reduced the incidence and pharyngeal colonization of serogroup C in general and that of ST11 in particular, with an almost complete absence of serogroup B ST11 isolates being observed [5]. Nevertheless, serogroup B ST11 has circulated with a certain frequency in Europe in the last few years. In the EU-IBIS database for 1999–2006, the ST11 complex/ET-37 complex represented 61.8% of the total number of serogroup C strains and 1.83% of serogroup B strains [17].

Using eBURST, 71 of the 107 STs identified in our region were classified in one group or lineage, with the remaining 36 STs being singletons. Twenty of these 36 singletons were previously included in one of the known clonal complexes, although with the stringent group definition of six of seven shared alleles their clonal relationship could not be established with this system. Despite its greater discriminatory power, eBURST allowed new clonal relationships to be identified, and two hitherto unidentified groups were established. Both groups were composed mainly (6/9) of STs described for the first time in this study. The number of alleles and the number of polymorphic sites at each locus provide complementary information on the diversity of the meningococcal population analyzed, allowing detection of the intracomplex or intragroup variation, which cannot be observed with isolated analysis of STs and clonal complexes.

The reduction in meningococcal circulation and pharyngeal colonization has been suggested to produce a niche that could be occupied by other isolates, possibly contributing to the diversity and expansion of new emerging clones [9]. A significant increase in the genetic diversity of invasive isolates using MLST and S.T.A.R.T. analysis was observed in a study performed in Scotland between 1999 and 2002 [10]. We found that the epidemic circulation of serogroup C ST8 isolates in 1997–1998 and ST11 isolates (serogroups B and C) in 2002–2004 increased clonality in these epidemic years. Nevertheless, the parameters used in this study show that the diversity of the population of invasive meningococci in our region did not increase from 1997–2008. In the present study, we identified 54 new STs, although these STs appeared continuously over time.

Despite two vaccination campaigns, the diversity of the population of invasive meningococci in the Basque Country remained fairly constant over time, with few changes in recombination phenomena, as shown by the I_A_ obtained over the 12 years, and the meningococcal population was always structured in stable clonal complexes.

Identification of new STs, the use of more precise tools for their classification, such as eBURST, and recognition of hitherto unidentified groups has great epidemiological value to analyze the evolution of the meningococcal population, not only in a discrete geographical region but also globally. The methods used for typing and classifying the population of meningococci analyzed in the present study were complementary. The combination of techniques with low and high discriminatory power allowed us to determine the evolution of the invasive meningococcal population over time, analyze its phenotypic and genotypic diversity, and the degree of genetic recombination among strains

In conclusion, the population of invasive meningococci in the Basque Country is basically similar to that in other European areas but shows certain regional distinguishing features. Vaccination in this region practically eliminated episodes of meningococcal C disease in vaccinated individuals but did not contain the activity of isolates of this serogroup in the non-vaccinated population and had little effect on the diversity of the invasive meningococcal population in the area.
